# BioWordVec, improving biomedical word embeddings with subword information and MeSH

**DOI:** 10.1038/s41597-019-0055-0

**Published:** 2019-05-10

**Authors:** Yijia Zhang, Qingyu Chen, Zhihao Yang, Hongfei Lin, Zhiyong Lu

**Affiliations:** 1 National Center for Biotechnology Information (NCBI), National Library of Medicine (NLM), National Institutes of Health (NIH), Bethesda, Maryland 20894 USA; 2 School of Computer Science and Technology, Dalian University of Technology, Dalian, Liaoning 116023 China

**Keywords:** Machine learning, Literature mining

## Abstract

Distributed word representations have become an essential foundation for biomedical natural language processing (BioNLP), text mining and information retrieval. Word embeddings are traditionally computed at the word level from a large corpus of unlabeled text, ignoring the information present in the internal structure of words or any information available in domain specific structured resources such as ontologies. However, such information holds potentials for greatly improving the quality of the word representation, as suggested in some recent studies in the general domain. Here we present BioWordVec: an open set of biomedical word vectors/embeddings that combines subword information from unlabeled biomedical text with a widely-used biomedical controlled vocabulary called Medical Subject Headings (MeSH). We assess both the validity and utility of our generated word embeddings over multiple NLP tasks in the biomedical domain. Our benchmarking results demonstrate that our word embeddings can result in significantly improved performance over the previous state of the art in those challenging tasks.

## Abstract (summary)

**Table:** 

Design Type(s)	data transformation objective • data integration objective • text processing and analysis objective
Measurement Type(s)	word representation
Technology Type(s)	Text_Mining
Factor Type(s)	
Sample Characteristic(s)

Machine-accessible metadata file describing the reported data (ISA-Tab format)

## Background & Summary

Distributed word representations learn dense and low-dimensional word embeddings from large unlabeled corpora and effectively capture the implicit semantics of words^1–3^. The low-dimensional word embedding is much more suitable for the recent neural-based deep learning models than the traditional one-hot representation. Based on word embeddings, the recent deep learning methods have been successfully applied to various natural language processing (NLP) tasks^4–6^. With the rapid advance in deep learning, word embeddings have become an integral part of NLP models and attracted significant attention.

In recent years, several word embedding models and pre-trained word embeddings^1,7,8^ have been made publicly available and successfully applied to many biomedical NLP (BioNLP) tasks. More recently, Wang *et al*. compared the performance of different word embeddings which were trained on four kinds of corpora including clinical notes, biomedical literature, Wikipedia and news articles^9^. Smalheiser *et al*. proposed a novel vector representation of words based on the similarity and co-occurrence frequency of words^10^.

However, the traditional biomedical word embeddings described above have two limitations. First, most of them were trained using the word2vec^1^ or GloVe model^7^, which uses a distinct vector to represent each word and ignores the internal structure of words. Such models are not particularly good at learning rare or out of vocabulary (OOV) words in the training data. If a word embedding model can capture the subword information and exploit the internal structure of words to augment the embedding representations in those rare or OOV words, it has the potential to greatly benefit various BioNLP applications. Bojanowski *et al*. recently proposed a novel embedding model^11^, which can effectively use the subword information to enrich the final word embedding results. In contrast to the word2vec model^1^, the subword embedding model makes use of the representations of character n-grams based on the unlabeled corpora, and then uses the sum of the n-gram vectors to represent the final word vector.

Moreover, existing word embedding models mainly focus on using the single source of large text corpora in PubMed and/or PubMed Central (PMC). Recently, some studies^12–15^ have suggested that integrating domain knowledge with the text corpora can be beneficial to improve the quality of word embeddings. In the biomedical domain, there are abundant biomedical knowledge data such as the medical subject headings (MeSH) and unified medical language system (UMLS), which could be explored to complement the textual information in the literature. Intuitively, integrating such biomedical domain knowledge should help improve the quality of word embedding such that it better captures the semantics of specialized terms and concepts.

In this work, we create BioWordVec: a new set of word vectors/embeddings using the subword embedding model on two different data sources: biomedical literature and domain knowledge in MeSH. Specifically, we construct a MeSH term graph based on the MeSH RDF data, followed by a random sampling strategy to generate a number of MeSH term sequences. Subsequently, we use the subword embedding model to learn the text sequences and MeSH term sequences in a unified n-gram embedding space. Our word embeddings are assessed for both validity and utility on multiple BioNLP tasks. As shown in our experimental results, our word embeddings outperform the current state-of-the-art word embeddings in all benchmarking tasks, suggesting that the subword information and domain knowledge is indeed able to improve the quality of biomedical word representations and better capture their semantics.

## Methods

**Fig. 1:**
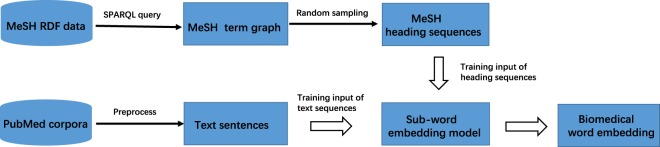
Schematic of learning word embedding based on PubMed literature and MeSH.

In this section, we present our method for learning biomedical word embeddings. This method consists of two steps: 1) constructing MeSH term graph based on its RDF data and sampling the MeSH term sequences and 2) employing the fastText subword embedding model to learn the distributed word embeddings based on text sequences and MeSH term sequences. A schematic overview of our method is shown in Fig. 1.

### Sampling MeSH term sequences

Recent studies (e.g.^16,17^) inspired by the skip-gram model^16,17^ have proposed to translate network/graphs into nodes sequences to learn networks embeddings. Similarly, in this work we transfer the relations of the MeSH term graph into ordered sequences of the heading nodes. This process results in main-heading sequences from MeSH and we subsequently combine them with PubMed sentence sequences for learning word embeddings.

There are two common sampling strategies: breadth-first sampling (BFS) and depth-first sampling (DFS). BFS gives the priority to sample the immediate neighbors of the source node, whereas DFS first samples the nodes as far as possible along each branch before backtracking. Grover *et al*.^18^ proposed a random walk procedure called node2vec that efficiently samples diverse neighborhoods in a network. In this work, we adopted this strategy to sample the sequences of main-heading nodes from the MeSH term graph. Specifically, let G, N and E denote the MeSH term graph, the node and edge set, respectively. A random walk is simulated to sample the sequence of a source node from G, which is guided by two parameters *p* and *q*. Let the random walk c starts with the node *u* (${c}_{0}=u$), and ${c}_{i-2}=t$, ${c}_{i-1}=v$ and ${c}_{i}=x$ denote the three continuous nodes *t*, *v* and *x* in the random walk. The generated distribution of *c*_*i*_ is defined as follow:

1 $$P\left({c}_{i}=x| {c}_{i-1}=v\right)=\left\{\begin{array}{ll}{\pi }_{vx} & if(v,x)\in E\\ 0 & otherwise\end{array}\right.$$

where *π*_*vx*_ is the transition probability from node *v* to *x*. The transition probability *π*_*vx*_ is defined as follows:

2 $${\pi }_{vx}=\alpha \left(t,x\right)=\left\{\begin{array}{ll}\frac{1}{p} & if\,{d}_{tx}=0\\ 1 & if\,{d}_{tx}=1\\ \frac{1}{q} & if\,{d}_{tx}=2\end{array}\right.$$

where *d*_*tx*_ is the shortest path between node *t* and *x*. Note that *d*_*tx*_ must be one of {0, 1, 2} because nodes *t*, *v* and *x* are three continuous nodes in a walk.

**Fig. 2:**
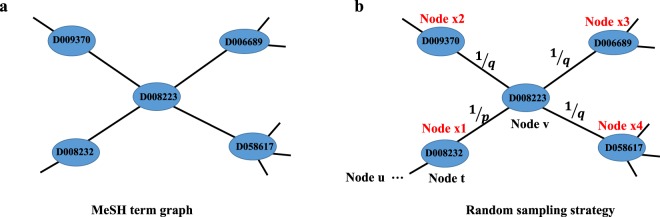
Illustration of the MeSH sequences sampling strategy. (**a**) An example of MeSH term graph. (**b**) Random sampling strategy.

In Fig. 2, we show an example MeSH term graph that contains five MeSH term nodes. Each MeSH term node is represented by its corresponding ID. The edges between the MeSH term nodes represent the relations between MeSH terms based on the MeSH RDF data. For example, the MeSH term nodes “D008232” and “D008223” represent MeSH headings “Lymphoproliferative Disorders” and “Lymphoma”, respectively. Based on the MeSH RDF data, the relation from MeSH headings “Lymphoproliferative Disorders” to “Lymphoma” is “meshv:broaderDescriptor”, which means that “Lymphoma” is included by the higher level heading “Lymphoproliferative Disorders”. In MeSH term graph, we simply use the undirected edges to represent the relations between MeSH terms, and do not distinguish the types and direction of the relations. Figure 2b illustrates the MeSH sequences sampling strategy based on the 2nd order random walk. Suppose the random walk starting with node *u* just traversed from node *t* (“D008232”) to node *v* (“D008223”), and we have four choices for the next step (“D008232”, “D009370”, “D006689” and “D058617”). For the node *x*_*1*_ (“D008232”), the transition probability is 1/*p* based on the equation (1) and (2), because the shortest path distance *d*_*tx*_ between node *t* (“D008232”) and node *x*_*1*_ (“D008232”) is 0. Similarly, the transition probability from node *v* (“D008223”) to node *x*_*2*_ (“D009370”), node *x*_*3*_ (“D006689”), node *x*_*4*_ (“D058617”) are all 1/*q*. Based on the equation (2), the two parameters *p* and *q* make the sampling strategy effectively combine the BFS and DFS. In our work, we applied this sampling strategy to simulate random walks starting from each main-heading node in MeSH and generated the MeSH term sequences. As a result, we transform the MeSH term graph into a set of sampling sequences.

A sequence sampled from the MeSH term graph is essentially an ordered set of MeSH main-heading nodes *D*_1_, *D*_2_, …, *D*_*l*_ where *l* is the sampling length parameter. For the sampling length, we empirically set as 100. Each MeSH term node is represented by its corresponding ID such as “D008232”, “D008223” and “D058617”. For example, “…, D008232, D008223, D058617, …” may be one of the MeSH term sequences sampled from Fig. 2a. Unlike previous studies such as^16–18^ that aim to learn embeddings for nodes, we transform MeSH IDs sequences into word sequences so that they can be treated equally as PubMed sentences during the learning of word embeddings. In this study, we directly used the text label of each MeSH ID in the MeSH RDF data (e.g. we use “lymphoproliferative disorders” for “D008232”). As a result, the list of MeSH IDs is turned into a text sequence consisting of words only.

### Subword embedding model

Bojanowski *et al*. proposed fastText: a subword embedding model^11^ based on the skip-gram model^1^ that learns the character n-grams distributed embeddings using unlabeled corpora where each word is represented as the sum of the vector representations of its n-grams. Compared to the word2vec model^1^, the subword embedding model can make effective use of the subword information and internal word structure to improve the embedding quality. In the biomedical domain, many specialized compound words, such as “deltaproteobacteria”, are rare or OOV in the training corpora, thus making them difficult to learn properly using the word2vec model. In contrast, the subword embedding model is naturally more suitable to deal with such situations. For instance, since “delta”, “proteo” and “bacteria” are common in the training corpora, the subword embedding model can learn the distributed representations of all character n-grams of “deltaproteobacteria”, and subsequently integrate the subword vectors to create the final embedding of “deltaproteobacteria”. In this study, we apply the subword embedding model to learn word embeddings from the joint text sequences of PubMed and MeSH.

The fastText subword embedding model^11^ is essentially a variant of the continuous skip-gram model^1^. Given a training word sequence *w*_1_, *w*_2_, …, *w*_*T*_, the objective function of the skip-gram model is defined as follow:

3 $$J=max\frac{1}{T}\sum _{1}^{T}\sum _{c\in {C}_{t}}logp({w}_{c}| {w}_{t})$$

where *C*_*t*_ is the set of the surrounding words of *w*_*t*_. Given the current word *w*_*t*_, the $p({w}_{c}| {w}_{t})$ is defined as the probability of observing its surrounding word *w*_*c*_.

4 $$p\left({w}_{c}| {w}_{t}\right)=\frac{{e}^{s\left({w}_{t},{w}_{c}\right)}}{{\sum }_{j=1}^{W}{e}^{s({w}_{t},{w}_{j})}}$$

where *s*(*w*_*t*_, *w*_*c*_) is the scoring function. The original skip-gram model defines the scoring function as scalar product, namely $s({w}_{t},{w}_{c})={u}_{wt}^{T}{v}_{wc}$, where *u*_*wt*_ and *v*_*wc*_ are the vectors of two words *w*_*t*_ and *w*_*c*_, respectively. This means the original skip-gram model can only learn a distinct vector for each word but cannot exploit subword information.

To address this issue, the subword embedding model represents a word as a bag of character n-grams. For example, the word “function” will be represented by the character 4 grams including <#fun, func, unct, ncti, ctio, tion, ion#> and the word itself <function>. The major difference between subword embedding model and the original skip-gram model is that the subword embedding model defines the *s*(*w*_*t*_, *w*_*c*_) as the ${\sum }_{g\in (1,\ldots ,G)}{z}_{g}^{T}{v}_{c}$, where (1, …, G) is the n-gram set of *w*_*t*_. *z*_*g*_ is the vector of character n-gram g, and *v*_*c*_ is the vector of word *w*_*c*_. Hence, the subword embedding model learns the distributed representation of character n-grams. Based on these n-grams representations, a word is represented as the sum of the vector representations of its n-grams. The advantage of the subword embedding model is that it shares the representations of n-grams across words, which is significantly helpful for learning reliable embedding for rare or OOV words.

In this work, the input of the subword model is the joint text sequences from PubMed and MeSH. For the PubMed text, the model aims to maximize the objective function ${J}_{PubMed}=\frac{1}{T}{\sum }_{1}^{T}{\sum }_{c\in {C}_{t}}logp({w}_{c}| {w}_{t})$, where T is the total vocabulary size. For the MeSH term sequences, the model aims to maximize the objective function ${J}_{MeSH}=\frac{1}{N}{\sum }_{1}^{N}{\sum }_{c\in {C}_{t}}logp({D}_{c}| {D}_{t})$, where N is the total number of MeSH main headings. We linearly combined the above two objective functions as follow:

5 $$J={J}_{PubMed}+{J}_{MeSH}$$

The training of our model is to maximize the above objective function in (5), which will learn the joint PubMed text sequences and MeSH term sequences in word embedding. Since both the PubMed sentence sequences and MeSH term sequences are word sequences, the subword embedding model can share the n-gram representations between the PubMed text words and MeSH term terms, thus integrating the PubMed and MeSH into a unified embedding space. The *J*_*PubMed*_ and *J*_*MeSH*_ are trained together by the subword embedding model.

### Implementation details

In our experiments, we downloaded the PubMed XML source files from https://www.nlm.nih.gov/databases/download/pubmed_medline.html. Our PubMed data contains 27,599,238 articles including the titles and abstracts. We extracted the title and abstract texts from the PubMed XML files to construct the PubMed text data. All words were converted to lowercase. The final PubMed text data contain 3,658,450,658 tokens.

For MeSH, we downloaded its RDF data (ftp://ftp.nlm.nih.gov/online/mesh/rdf/) on 3/19/2018. The MeSH terms consist of descriptor terms, qualifier terms and supplementary concept record terms. The MeSH descriptor terms are known as main headings for describing the core subjects of a PubMed article. Thus in this study, we focus on the MeSH descriptors terms. Note that it is not straightforward to handle punctuation marks such as comma in MeSH descriptor terms given their different uses. For example, comma is used differently between D001990 (“Bronchiolitis, Viral”) and D013676 (“Technology, Industry, and Agriculture”). Hence in this work, we simply removed them from the MeSH descriptor terms (this pre-processing step is to be improved in the future but is beyond the scope of this work) and converted the words to lowercase. We used SPARQL queries to retrieve the relations between descriptors terms from the MeSH RDF data, resulting in a MeSH term graph with 28,436 main heading nodes and 52,013 relations. For each main heading node, we sampled 10 MeSH term sequences, resulting in a total of 284,360 MeSH term sequences.

Our word embeddings were trained by the following hyper-parameters empirically. For the sampling strategy, the two parameters *p* and *q* were set as 2 and 1, respectively. For each node in MeSH term graph, we sampled 10 sequences of fixed length (*l* = 150). The dimension of the word vectors was set to be 200, and the size of the negative sample size was set to be 10. Similar to Bojanowski *et al*.^11^, all n-grams (3 ≤ *n* ≤ 6) were extracted by the subword model for training word representations.

## Data Records

Word embeddings are commonly used and evaluated in two types of (Bio-)NLP tasks: intrinsic and extrinsic. For intrinsic tasks, word embeddings are used to calculate or predict semantic similarity between words, terms or sentences. For extrinsic tasks, word embeddings are used as the input for various downstream NLP tasks, such as relation extraction or text classification. Chiu *et al*.^8^ suggested that the extrinsic tasks benefit from smaller window size while the opposite the intrinsic tasks. In our preliminary experiments, we also observed similar results: when setting the context window size as 20 and 5, our word embedding achieved the highest performance in intrinsic and extrinsic evaluation, respectively. Hence in this work, we followed their lead and created two specialized, task-dependent sets of word embeddings via setting the context window size as 20 and 5, respectively. Our BioWordVec data are freely available on Figshare^19^. Both sets are in binary format and contain 2,324,849 distinct words in total where 2,309,172 words come from the PubMed and 15,677 from MeSH. All words were converted to lowercase and the number of dimensions is 200.

Our word embeddings can effectively integrate the MeSH term sequences to improve the representation of such terms or concepts. In Table 1, we show a randomly selected set of term pair examples from the manually-annotated UMNSRS-Sim^20^ and UMNSRS-Rel^20^ datasets and calculated the cosine similarity of the term pairs. It can be seen that all term pairs in Table 1 have relatively high scores from both UMNSRS-Sim^20^ and UMNSRS-Rel^20^. For a good word embedding method, it should yield similarly high cosine similarity scores for these pairs. Table 1 shows that the cosine similarity score calculated by our word embedding is higher than the other word embeddings^1,8,11,21^. For example, the cosine similarity between “mycosis” and “histoplasmosis” is 0.353, 0.544, and 0.595 by Mikolov *et al*.^1^, Pyysalo *et al*.^21^ and Chiu *et al*.^8^, respectively, but 0.706 by our word embedding. On the other hand, it is difficult to determine how high their absolute cosine similarity score should be. Hence, we further performed technical validation using the Pearson’s correlation coefficient and Spearman’s correlation coefficient in the following Section.

**Table 1:** The cosine similarity of the word pair examples by different word embeddings.

Word pair	UMNSRS-Sim^20^	UMNSRS-Rel^20^	Mikolov *et al*.^1^	Pyysalo *et al*.^21^	Chiu *et al*.^8^	BioWordVec (win 20)
thalassemia, hemoglobinopathy	1307	1218	—	0.713	0.754	0.834
mycosis, histoplasmosis	1137.25	1185.75	0.353	0.544	0.595	0.706
thirsty, hunger	935.75	1249	0.252	0.425	0.59	0.629
influenza, pneumoniae	898.5	1354	0.482	0.252	0.514	0.611
atherosclerosis, angina	936	1357.75	0.503	0.506	0.506	0.589

“win20” denotes the BioWordVec was trained by setting the context window size as 20. “UMNSRS-Sim^20^” and “UMNSRS-Rel^20^” denote the mean score of the word pair from UMNSRS-Sim^20^ and UMNSRS-Rel^20^.

In particular, our word embeddings can make good use of the sub-word information and internal structure of words to improve the representations of the rare words, which is highly valuable for BioNLP applications. For example, the word “deltaproteobacteria” is a rare word even in the biomedical corpus. In Table 2, we gave the top 5 most similar words of “deltaproteobacteria” by our method vs. Chiu *et al*.^8^. It can be seen that our method capture the similar words of “deltaproteobacteria” better than Chiu *et al*.^8^. Due to the common sub-words “proteo” and “bacteria”, our method can easy capture the similar words such as “betaproteobacteria” and “zetaproteovacteria”.

**Table 2:** The top 5 most similar words of “deltaproteobacteria”.

BioWordVec (win20)		Chiu *et al*.^8^	
Top 5 similar words	Similarity score	Top 5 similar words	Similarity score
deltaproteobacterial	0.985	magnetospirilla	0.861
deltaproteobacterium	0.963	Thermales	0.857
betaproteobacteria	0.952	Acidiphilium-like	0.854
zetaproteobacteria	0.945	nirK1	0.85
delta-proteobacteria	0.939	nostoc	0.847

“win20” denotes the BioWordVec was trained by setting the context window size as 20.

## Technical Validation

To validate our method, two widely-used benchmarking datasets UMNSRS-Sim^20^ and UMNSRS-Rel^20^ were employed. UMNSRS-Sim and UMNSRS-Rel respectively consist of 566 and 587 term pairs, with their corresponding relatedness/similarity scores manually judged by the domain experts from the University of Minnesota Medical School. For evaluation, we first use word embeddings to calculate a cosine similarity score for each term pair. Then, we measure the Pearson’s correlation coefficient and Spearman’s correlation coefficient between the computed scores and those provided by human experts. We compared our method with several state-of-the-art methods^1,8,11,21^. Mikolov *et al*.^1^ proposed the word2vec model and provided the pre-trained word embeddings on Google news. Using the same word2vec model, Chui *et al*.^8^ and Pyysalo *et al*.^21^ provide biomedical word embeddings based on PubMed and PubMed Central articles.

Our method has two variants: One was trained with only PubMed data, and the other using both PubMed and MeSH data. From Table 3, it can be seen that our method significantly outperforms the other methods. The results suggest that the subword information and MeSH data are valuable and helpful in biomedical domain. We also noticed that the biomedical corpus was more suitable in this case than the general English corpus. Both Mikolov *et al*.^1^ and Pyysalo *et al*.^21^ used the same word2vec model and default parameters, but the word embeddings trained on PubMed and PMC corpus significantly outperformed the ones trained by Google news in our results.

**Table 3:** Evaluation results on UMNSRS datasets.

Method	Corpus	UMNSRS-Sim			UMNSRS-Rel		
		#	Pearson	Spearman	#	Pearson	Spearman
Mikolov *et al*.^1^	Google news	336	0.421	0.409	329	0.359	0.347
Pyysalo *et al*.^21^	PubMed + PMC	493	0.549	0.524	496	0.495	0.488
Chiu *et al*.^8^	PubMed	462	0.662	0.652	467	0.600	0.601
BioWordVec (win20)	PubMed	**521**	0.665	0.654	**532**	0.608	0.607
BioWordVec (win20)	PubMed + MeSH	**521**	**0.667**	**0.657**	**532**	**0.619**	**0.617**

“#” denotes the number of the term pairs that can be mapped by the different word embeddings. “Pearson” and “Spearman” denote the Pearson’s correlation coefficient score and Spearman’s correlation coefficient score, respectively. “win20” denotes the BioWordVec was trained by setting the context window size as 20. The highest value is shown in bold.

In Table 3, we show that our method achieves better performance on both datasets. Chui *et al*.^8^ also achieves competitive performance which significantly outperforms Mikolov *et al*.^1^ and Pyysalo *et al*.^21^. In Table 3, the correlation results are not directly comparable because the approaches were evaluated on different sets of term pairs. Hence, we show in Table 4 the results on the common set of term pairs found by both our method and Chui *et al*.^8^, which include 459 and 461 pairs in UMNSRS-Sim and UMNSRS-Rel, respectively. It can be seen that the performance improvement is greater by our method on these two common sets.

**Table 4:** Comparison results on UMNSRS datasets using the common term pairs.

Method	Corpus	UMNSRS-Sim			UMNSRS-Rel		
		#	Pearson	Spearman	#	Pearson	Spearman
Chiu *et al*.^8^	PubMed	459	0.661	0.651	461	0.600	0.601
BioWordVec (win20)	PubMed	459	0.679	0.665	461	0.624	0.626
BioWordVec (win20)	PubMed + MeSH	459	**0.681**	**0.668**	461	**0.633**	**0.635**

“#” denotes the number of the term pairs that can be mapped by the different word embeddings. “Pearson” and “Spearman” denote the Pearson’s correlation coefficient score and Spearman’s correlation coefficient score, respectively. “win20” denotes the BioWordVec was trained by setting the context window size as 20. The highest value is shown in bold.

## Usage Notes

We demonstrate here the application of BioWordVec in two separate use cases: finding similar sentences and extracting biomedical relations.

### Use case 1: sentence pair similarity

Word embeddings are often used to calculate sentence pair similarity^22^. In the general domain, the SemEval Semantic Textual Similarity (SemEval STS) challenge has been organized for over five years, which calls for effective models to measure sentence similarity^23^. Averaged word embeddings are used as a baseline to measure sentence pair similarity in the challenges: each sentence is transformed as a vector by averaging the word vectors for each word in the sentence and sentence pair similarity is effectively measured by the similarity between the averaged vectors using common measures such as Cosine and Euclidean similarity.

Sentence similarity is also critical in biomedical and clinical domains^24,25^. We conducted a case study to quantify the effectiveness of the proposed embeddings in the task of computing sentence pair similarity on clinical texts. We used the BioCreative/OHNLP STS dataset, which consists of 1,068 pairs of sentences derived from clinical notes and were annotated by two medical experts on a scale of 0–5, from completely dissimilar to semantically equivalent^26^. The top-ranked submission model used average embeddings with different similarity functions, which was shown effective to capture sentence similarity^27^. We applied averaged word embedding approach and adopted Cosine, Euclidean and City Block similarity to measure the averaged vectors. The result was evaluated based on Pearson’s Correlation between the predicted similarities and gold standard labels.

Table 5 shows the evaluation results on clinical sentence pair similarity. Our proposed embeddings achieved higher correlations in all three similarity measures. This demonstrates that the proposed embeddings more effectively capture the semantic meaning. On the other hand, we also noted that “BioWordVec (win20) w/o MeSH” and “BioWordVec (win20) w/MeSH” achieved similar correlation scores in all three similarity measures, which indicates integrating MeSH was not much helpful in this task. It is likely because MeSH plays a more vital role in PubMed articles than in clinical notes.

**Table 5:** Sentence pair similarity results on BioCreative/OHNLP STS dataset.

Similarity measures	Mikolov *et al*.^1^	Pyysalo *et al*.^21^	Chiu *et al*.^8^	BioWordVec (win20) w/o MeSH	BioWordVec (win20) w/MeSH
Cosine	0.768	0.755	0.757	0.770	**0.771**
Euclidean	0.725	0.723	0.727	0.751	**0.753**
Block	0.725	0.722	0.727	0.750	**0.752**

“win20” denotes the BioWordVec was trained by setting the context window size as 20.

### Use case 2: biomedical relation extraction

Given that word embeddings are often used as the input in recent deep-learning based methods for various biomedical NLP tasks^4–6^, below we evaluate its effect in some biomedical relation extraction tasks. In our experiments, we evaluate its effect in two biomedical relation extraction tasks: protein-protein interaction (PPI) extraction and drug-drug interaction (DDI) extraction, respectively. The former is a binary relation extraction task, whereas the latter a multi-class relation extraction task. Following previous studies^4,28^, we use precision, recall and F-score as evaluation metrics and choose the same baseline methods.

The public PPI corpora were used for the PPI extraction, including AIMed^29^, BioInfer^30^, IEPA^31^, HPRD50^32^ and LLL^33^. The detailed statistics of the PPI corpora is listed in Table 6. For this binary relation extraction task, we implemented a convolutional neural network (CNN) model and used the dropout layer with a dropout rate of 0.5 after the embedding layer and before the output layer. For the input of our CNN model, we combine the position embeddings with the word embeddings as they have been shown to be effective^34^. The PPI extraction experiments were evaluated with 10-fold document-level cross validation. As shown in Table 7, our method achieves the highest F-score on all datasets except one (Chiu *et al*.^8^ achieved higher F-score than our method on AIMed). Knowledge from MeSH was helpful in all datasets.

**Table 6:** The statistics of the PPI corpora.

Corpus	Sentences	Positive	Negative	Total
AIMed	1955	1,000	4,834	5,834
BioInfer	1100	2,534	7,132	9,666
IEPA	145	335	482	817
HPRD50	486	163	270	433
LLL	77	164	166	300

**Table 7:** PPI extraction evaluation results on five PPI corpora.

Data Set	Mikolov *et al*.^1^		Pyysalo *et al*.^21^		Chiu *et al*.^8^		BioWordVec (win5) w/o MeSH		BioWordVec (win5) w/MeSH	
	F-Score	*σ*	F-Score	*σ*	F-Score	*σ*	F-Score	*σ*	F-Score	*σ*
AIMed	0.445	0.076	0.457	0.087	**0.492**	0.064	0.484	0.101	0.487	0.081
BioInfer	0.524	0.038	0.532	0.044	0.545	0.053	0.543	0.041	**0.549**	0.039
IEPA	0.603	0.062	0.597	0.062	0.615	0.061	0.617	0.049	**0.623**	0.064
HPRD50	0.484	0.187	0.499	0.121	0.481	0.145	0.504	0.136	**0.511**	0.13
LLL	0.679	0.12	0.688	0.093	0.684	0.124	0.708	0.092	**0.713**	0.095

The highest value is shown in bold. “*σ*” denotes the standard deviation of the F-score. “win5” denotes the BioWordVec was trained by setting the context window size as 5.

For the DDI extraction, we used the DDI 2013 corpus^35,36^, which is manually annotated and consists of five different DDI types, including *Advice*, *Effect*, *Mechanism*, *Int* and *Negative*. Since the DDI extraction is a multi-class relation extraction task, we compute the micro average to evaluate the overall performances^37,38^. In the DDI 2013 corpus, the training set and test set contain 27,792 and 5,716 instances, respectively. We randomly split 10% of the training data as the method validation set and report the results on the test set.

In the DDI extraction experiments, we first applied the same CNN model as the PPI experiments. To further evaluate the performance of different word embeddings on more complex neural models, we also conducted a comparison experiment using a recent state-of-the-art DDI extraction model^4^ which is a hierarchical RNNs with a input attention layer based on the sentence sequence and shortest dependency path.

The experimental results in Table 8 show that our method achieved the highest F-score on both CNN and RNN models. We also noticed that our method achieved more significant advantage on the simple CNN model than the complex RNN model. For example, our method and Mikolov *et al*.^1^ achieved the F-score of 0.687 and 0.636 on CNN model, respectively. The advantage of F-score between our method and Mikolov *et al*.^1^ was more than 0.05. When employing the state-of-the-art RNN model, the improvement of F-score reduces to 0.033. This is likely due to the fact that the state-of-the-art DDI extraction model^4^ already integrates the shortest dependency path information and part-of-speech embedding, as well as using the multiple layer of bidirectional long short-term memory networks (LSTMs) to boost the performance. Taken together, these complex steps/strategies partly reduced the importance of the word embedding for the DDI extraction task.

**Table 8:** DDI extraction evaluation results on DDI 2013 corpus.

Method	Corpus	CNN model			hierarchical RNN model		
		Precision	Recall	F-score	Precision	Recall	F-score
Mikolov *et al*.^1^	Google news	0.698	0.584	0.636	0.681	0.699	0.691
Pyysalo *et al*.^21^	PubMed + PMC	0.689	0.624	0.655	0.692	**0.727**	0.709
Chiu *et al*.^8^	PubMed	**0.709**	0.650	0.677	0.749	0.691	0.719
BioWordVec (win5)	PubMed	0.696	0.669	0.683	0.744	0.702	0.722
BioWordVec (win5)	PubMed + MeSH	0.694	**0.679**	**0.687**	**0.757**	0.696	**0.724**

The highest value is shown in bold. “win5” denotes the BioWordVec was trained by setting the context window size as 5.

## ISA-Tab metadata file

Download metadata file

## Data Availability

The source code for generating BioWordVec is freely available at https://github.com/ncbi-nlp/BioWordVec. The PubMed data are available from https://www.nlm.nih.gov/databases/download/pubmed_medline.html. The MeSH RDF data are available from https://www.nlm.nih.gov/databases/download/mesh.html.
